# Concurrent Immunoglobulin Light Chain and Transthyretin Cardiac Amyloidosis Patient Treated With Daratumumab and Tafamidis: A Case Report

**DOI:** 10.1002/jha2.70122

**Published:** 2025-08-13

**Authors:** Taiki Nishihara, Yawara Kawano, Masayoshi Tasaki, Ayuko Naito, Hiromichi Yuki, Yasuhiro Nagayoshi, Seiji Takashio, Nao Nishimura, Mitsuharu Ueda, Kenichi Tsujita, Jun‐Ichirou Yasunaga

**Affiliations:** ^1^ Department of Cardiovascular Medicine Amakusa Medical Center Amakusa Japan; ^2^ Department of Hematology Rheumatology, and Infectious Diseases Graduate School of Medical Sciences Kumamoto University Kumamoto Japan; ^3^ Department of Neurology Graduate School of Medical Sciences Kumamoto University Kumamoto Japan; ^4^ Department of Clinical Biosciences Graduate School of Medical Sciences Kumamoto University Kumamoto Japan; ^5^ Department of Hematology Amakusa Medical Center Amakusa Japan; ^6^ Department of Cardiovascular Medicine Graduate School of Medical Sciences Kumamoto University Kumamoto Japan

**Keywords:** AL amyloidosis, ATTR amyloidosis, case report, daratumumab, tafamidis

## Abstract

Immunoglobulin light chain (AL) and wild‐type transthyretin (ATTRwt) amyloidosis, while sharing similar clinical presentations, require distinct treatments. We report a rare case of a 75‐year‐old man with heart failure diagnosed with concurrent AL and ATTRwt cardiac amyloidosis. Immunohistochemistry and liquid chromatography‐tandem mass spectrometry confirmed both AL and ATTR amyloid deposits in the heart. The patient was safely and effectively treated with daratumumab‐based chemotherapy followed by tafamidis. This case underscores the necessity of precise amyloid typing for tailored therapy, suggesting sequential daratumumab and tafamidis as a viable strategy for this complex overlap.

**Trial Registration**: The authors have confirmed clinical trial registration is not needed for this submission.

## 1 Introduction

Immunoglobulin light chain (AL) and transthyretin (ATTR) amyloidosis are two common systemic amyloidoses often affecting the heart, sharing similar clinical presentations [1]. Recent advances in treatment for these two amyloidoses have led to an improvement in prognosis. However, treatments differ according to the type of amyloid (AL or ATTR). Therefore, accurate diagnosis, including amyloid typing, is critical for patient management. We report a rare case of AL and ATTR cardiac amyloidosis presenting in a single patient, successfully treated with the CD38 antibody, daratumumab, and the TTR tetramer stabilizer, tafamidis.

## 2 Case Presentation

**FIGURE 1:**
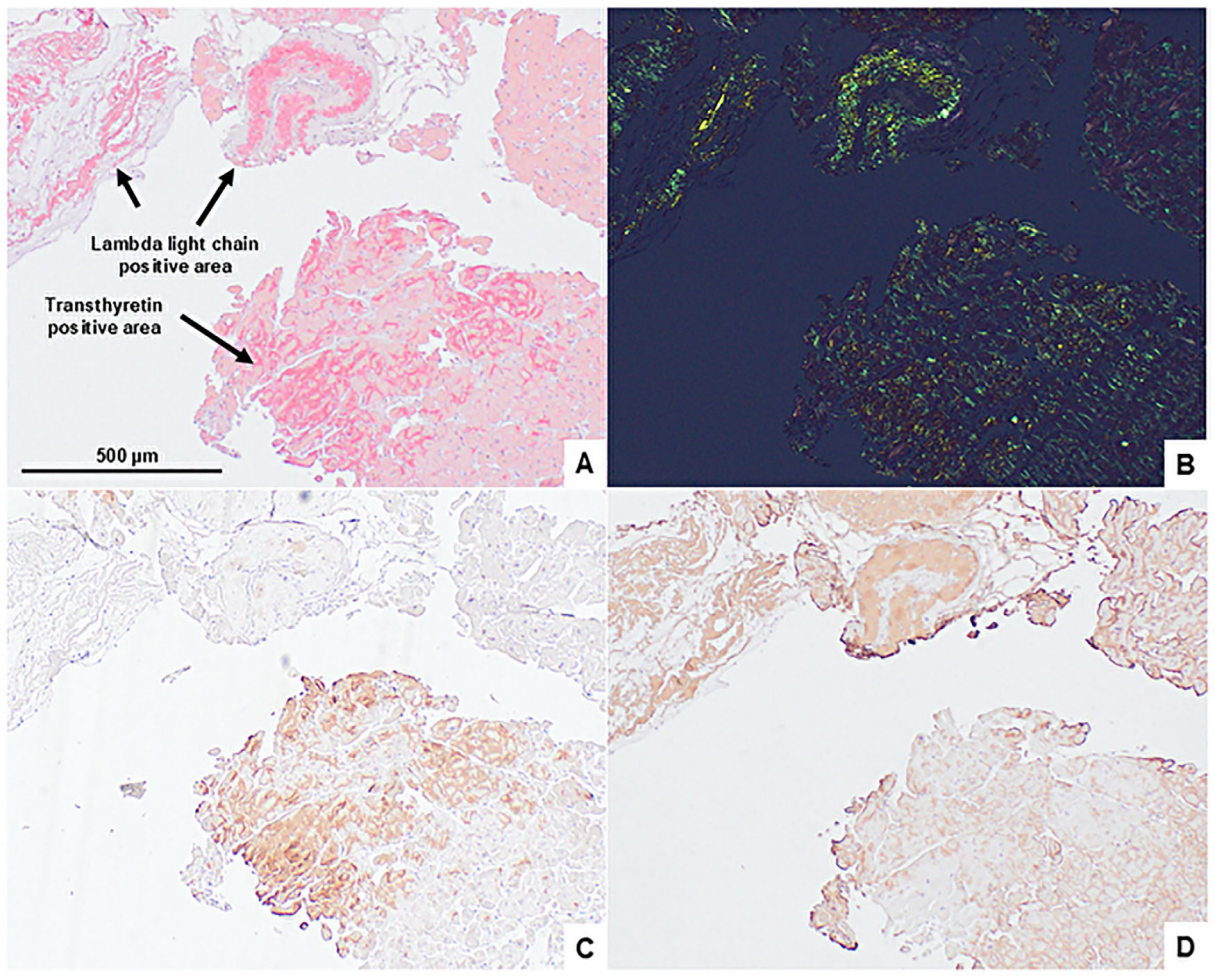
Histopathological staining of specimens obtained via endomyocardial biopsy. Congo red stained specimen visualized by light microscopy (A) and cross‐polarized light microscopy (B). Immunohistochemical stained specimen for expression of transthyretin (C) and lambda light chains (D).

**FIGURE 2:**
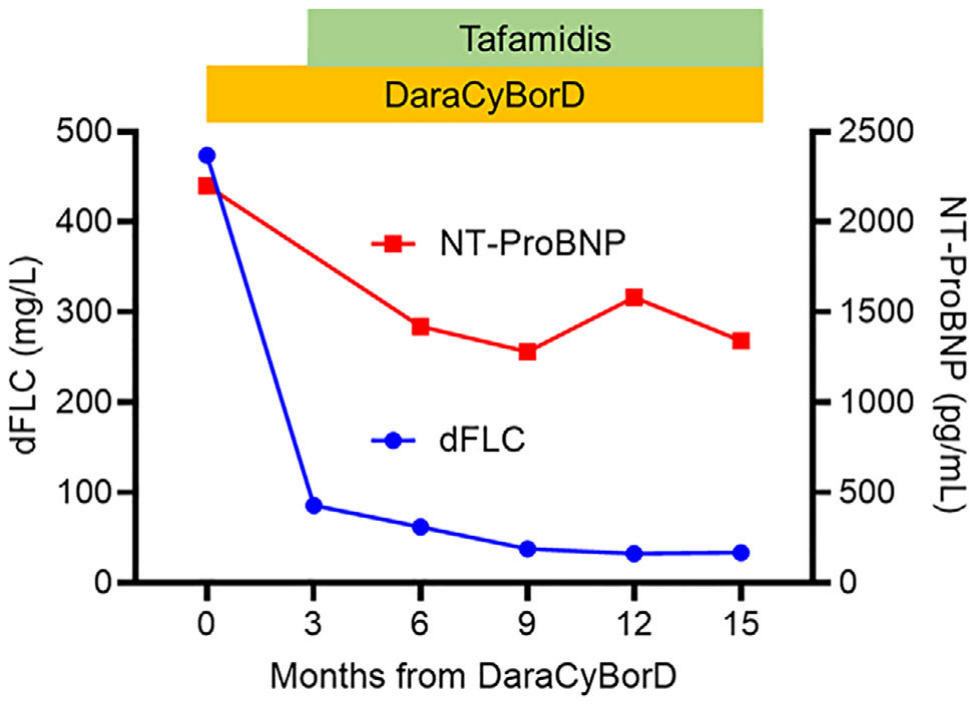
Change in dFLC and NT‐ProBNP levels according to treatments. dFLC: difference between involved and uninvolved free light chains, NT‐ProBNP: N‐terminal pro‐brain natriuretic peptide, DaraCyBorD: daratumumab, cyclophosphamide, bortezomib and dexamethasone

A 75‐year‐old man was presented to our hospital with shortness of breath and fatigue lasting for several days. He had edema of the lower legs with an increase of body weight by 4.8 kg in the past 3 months. The patient was considered to have heart failure (HF), classified as New York Heart Association (NYHA) functional class III. An electrocardiogram (ECG) showed bradycardic atrial fibrillation (AF) with a low QRS voltage in the limb leads with a heart rate of approximately 40 bpm. Chest radiography showed enlarged cardiac shadow, bilateral pleural effusion, and pulmonary congestion. Transthoracic echocardiography (TTE) revealed a left ventricular (LV) ejection fraction (EF) of 63%, LV wall thickness (17 mm at maximum), diastolic dysfunction (E/eʹ ratio of 13), and reduced longitudinal strain with apical sparing. During hospitalization, the patient's HF worsened, associated with bradycardic AF, leading to pacemaker implantation. Due to pacemaker implantation, cardiac MRI could not be performed. Based on his history of carpal tunnel syndrome, TTE findings, and grade‐2 cardiac uptake on ^99m^Tc‐pyrophosphate scintigraphy scan, the cause of his HF was suspected to be cardiac ATTR amyloidosis. However, blood tests showed an elevated serum lambda free light chain level at 516 mg/L (difference between involved and uninvolved free light chains [dFLC] 430 mg/L), accompanied by urine lambda‐type Bence‐Jones protein. Bone marrow examination revealed 9.2% of monoclonal plasma cells with *t*(11;14) translocation, which indicated the presence of AL amyloidosis. For precise diagnosis, immunohistochemical (IHC) analysis of the endomyocardial biopsy specimens was performed [2]. As a result, Congo red‐stained amyloid deposits with positive staining against transthyretin (TTR) and immunoglobulin lambda light chain (LLC) antibodies were identified (Figure 1). Moreover, the two distinct areas were separately captured and analyzed by laser capture microdissection combined with liquid chromatography tandem mass spectrometry (LMD‐LC‐MS/MS) [2]. Consequently, TTR and LLC were the main amyloidogenic proteins in the IHC‐positive lesions of TTR and LLC (Table 1). No amyloid deposits were detected in the gastric, duodenal, or bone marrow biopsy specimens. Additionally, there were no signs of amyloid involvement in other organs, such as the kidneys or liver. After confirming the negativity of mutations in the TTR gene, the patient was diagnosed with concurrent light chain (AL) (Mayo 2012 stage IV) [3] and wild‐type ATTR (ATTRwt) (Columbia score 5) [4] cardiac amyloidosis. The patient was first treated with daratumumab, cyclophosphamide, bortezomib, and dexamethasone (DaraCyBorD) [5] against AL amyloidosis, followed by oral tafamidis [6] administration against ATTRwt amyloidosis three months after DaraCyBorD initiation. In the DaraCyBorD regimen, subcutaneous daratumumab was administered at the standard dose, whereas the doses of cyclophosphamide (200 mg/m^2^/week), bortezomib (0.7 mg/m^2^/week), and dexamethasone (20 mg/week) were attenuated in consideration of the patient's advanced age and compromised cardiac function. Hematological partial response (PR) was achieved after three cycles of DaraCyBorD and very good partial response (VGPR) after Cycle 9, according to reduction of dFLC [7] (Figure 2). More than 30% of improvement in N‐terminal pro‐brain natriuretic peptide (NT‐ProBNP) (i.e., cardiac response) [7] was obtained after six cycles of DaraCyBorD (3 months from tafamidis administration) and has been maintained up to 15 months from treatment initiation (Figure 2). For the past 12 months of combination therapy with DaraCyBorD and tafamidis, no treatment‐related toxicities leading to hospitalization or discontinuation of the therapies were observed. Additionally, there had been no HF‐related hospitalizations, worsening of NYHA classification (currently class II), nor an increase in high‐sensitivity cardiac troponin T (hs‐cTnT) levels.

**TABLE 1:** List of proteins detected by liquid chromatography tandem mass spectrometry.

LLC positive region			TTR positive region		
Protein	Number of peptides	Relative value	Protein	Number of peptides	Relative value
Immunoglobulin lambda constant 2	94	0.111	Transthyretin	201	0.087
Apolipoprotein A‐IV	135	0.043	Actin, alpha skeletal muscle	174	0.029
Apolipoprotein E	87	0.034	Actin, alpha cardiac muscle 1	164	0.028
Apolipoprotein A‐I	63	0.03	Myosin regulatory light chain 2, ventricular/cardiac muscle isoform	61	0.023
Vitronectin	84	0.022	Myosin light chain 3	67	0.022
Serum amyloid P‐component	39	0.022	Actin, cytoplasmic 1	114	0.019
Prolargin	58	0.019	Myosin‐7	558	0.018
Clusterin	64	0.018	Desmin	109	0.015
Transthyretin	20	0.017	Tropomyosin alpha‐1 chain	64	0.014
Vimentin	58	0.016	Serum amyloid P‐component	44	0.013

Abbreviations: LLC: lamda light chain, TTR: transthyretin.

## 3 Discussion

We treated a rare case of concurrent AL and ATTRwt cardiac amyloidosis by novel therapeutic modalities, including a CD38 monoclonal antibody, daratumumab, and a TTR tetramer stabilizer, tafamidis. Precise subtyping of amyloid fibrils is crucial, since treatments and prognoses differ according to each subtype. Amyloid cardiomyopathy caused by ATTRwt amyloidosis may sometimes be difficult to distinguish from that caused by AL amyloidosis. Additionally, ATTR amyloidosis often coexists with MGUS [8], and therefore, differentiating ATTR from AL amyloidosis is of clinical importance. In the present case, IHC and LMD‐LC‐MS/MS were useful for not only determining the type of amyloid precursor proteins but also for identifying differences in co‐deposited proteins of AL and ATTR amyloidosis regions. A previous report analyzed differences in deposited proteins between cardiac AL and ATTR amyloidosis samples from different individuals [9]. However, there is always a possibility that differences in patient background may influence the outcomes of these data. Further analysis of concurrent AL and ATTR amyloidosis LMD‐LC‐MS/MS data, sharing the same individual background, can be useful for understanding the mechanisms and pathogenesis of each amyloidosis. Moreover, precise diagnosis led to rapid therapeutic intervention against AL amyloidosis of Mayo 2012 Stage IV, which is considered high risk of early mortality [3]. Since amyloidogenic immunoglobulin light chains themselves are known to have cardiac toxicity [10], it is important to make a correct diagnosis of cardiac AL amyloidosis by IHC and LMD‐LC‐MS/MS as early as possible, start DaraCyBorD, and reduce the toxic light chains as much as possible. We previously reported that increased hs‐cTnT levels 1 year after tafamidis administration were associated with worse clinical outcomes in ATTRwt cardiac amyloidosis [11]. In the current case, it is of clinical significance that there was no increase in hs‐cTnT level post 1 year of tafamidis treatment under the DaraCyBorD combination.

Although concurrent amyloidosis cases have been reported [12, 13], to our best knowledge, studies of concurrent AL and ATTRwt amyloidosis treated with novel agents are limited. We successfully treated the patient by adding a TTR tetramer stabilizer after CD38 antibody‐containing chemotherapy. The treatment has been effective and well‐tolerated in a relatively aged patient receiving pacemaker implantation with severe HF. Since therapeutic agents such as bortezomib and dexamethasone may cause cardiotoxicity [14], we added tafamidis after confirming the lack of toxicity and achievement of hematological response by DaraCyBorD. We suggest that sequential administration of DaraCyBorD and tafamidis is the key for safe treatment continuation. Although long‐term follow‐up is essential for identifying late toxicities and cardiac outcomes, we believe that the combination of daratumumab with tafamidis is an effective and feasible therapeutic option for concurrent AL and ATTR cardiac amyloidosis patients.

## Author Contributions

T.N. and Y.K. reviewed clinical data and wrote the paper. M.T. and M.U. conducted IHC and LMD‐LC‐MS/MS. T.N., Y.K., A.N., H.Y., Y.N., S.T., N.N., K.T., and J.Y. contributed to patient care and data collection. All authors approved the final version of the manuscript.

## Ethics Statement

The authors have nothing to report.

## Consent

Informed consent was obtained from the patient for publication of the manuscript.

## Conflicts of Interest

Yawara Kawano has received honoraria from Janssen Pharmaceuticals Inc. outside the submitted work. Masayoshi Tasaki has received grants from Pfizer outside the submitted work. Mitsuharu Ueda has received grants, personal fees, and non‐financial support from Pfizer outside the submitted work. Seiji Takashio has received honoraria from Pfizer outside the submitted work. Kenichi Tsujita has received honoraria from Pfizer outside the submitted work.

## Data Availability

The data related to this report will be made available from the corresponding author upon reasonable request.
